# A Study of LoRa: Long Range & Low Power Networks for the Internet of Things

**DOI:** 10.3390/s16091466

**Published:** 2016-09-09

**Authors:** Aloÿs Augustin, Jiazi Yi, Thomas Clausen, William Mark Townsley

**Affiliations:** 1 École polytechnique Route de Saclay, 91128 Palaiseau, France; aloys.augustin@gmail.com (A.A.); thomas.clausen@polytechnique.edu (T.C.); 2 Cisco Paris Innovation and Research Laboratory (PIRL), 11 Rue Camille Desmoulins, 92782 Issy les Moulineaux, France; townsley@cisco.com

**Keywords:** LoRa, Internet of Things, long range, low power

## Abstract

LoRa is a long-range, low-power, low-bitrate, wireless telecommunications system, promoted as an infrastructure solution for the Internet of Things: end-devices use LoRa across a single wireless hop to communicate to gateway(s), connected to the Internet and which act as transparent bridges and relay messages between these end-devices and a central network server. This paper provides an overview of LoRa and an in-depth analysis of its functional components. The physical and data link layer performance is evaluated by field tests and simulations. Based on the analysis and evaluations, some possible solutions for performance enhancements are proposed.

## 1. Introduction

The essential difference between “the Internet” and “the Internet of Things” (IoT) [1] is that in the IoT, there is just “less of everything” available in a given device or network device: less memory, less processing power, less bandwidth, etc.; and of course, less available energy. This is either because “things” are battery driven and maximizing lifetime is a priority or because their number is expected to be massive (it is estimated that there will be 50 billion connected devices by 2020 [2]). This drive to “do more with less” leads to constraints that limit the applicability of traditional cellular networks, as well as of technologies, such as WiFi, due to energy and scalability requirements.

Another range of protocols and technologies has emerged to fulfill the communication requirements of the IoT: Low-Power Wide Area Networks (LPWAN). Colloquially speaking, an LPWAN is supposed to be to the IoT what WiFi was to consumer networking: offering radio coverage over a (very) large area by way of base stations and adapting transmission rates, transmission power, modulation, duty cycles, etc., such that end-devices incur a very low energy consumption due to their being connected.

LoRa (LoRa Alliance, https://lora-alliance.org) is one such LPWAN protocol and the subject of study for this paper. LoRa targets deployments where end-devices have limited energy (for example, battery-powered), where end-devices do not need to transmit more than a few bytes at a time [3] and where data traffic can be initiated either by the end-device (such as when the end-device is a sensor) or by an external entity wishing to communicate with the end-device (such as when the end-device is an actuator). The long-range and low-power nature of LoRa makes it an interesting candidate for smart sensing technology in civil infrastructures (such as health monitoring, smart metering, environment monitoring, etc.), as well as in industrial applications.

### 1.1. Related Work

Different communication technologies aimed at low power, wireless IoT communication have been proposed and deployed. As indicated above, these grossly fall within two categories:

- Low power local area networks with a less than 1000-m range. This category includes IEEE 802.15.4, IEEE P802.1ah, Bluetooth/LE, etc., which are applicable directly in short-range personal area networks, in body area networks or, if organized in a mesh topology, also in larger areas.
- Low-power wide area networks, with a greater than 1000-m range, essentially low-power versions of cellular networks, with each “cell” covering thousands of end-devices. This category includes LoRaWAN, but also protocols, such as Sigfox, DASH7, etc.

This section provides a perspective on LoRaWAN by giving a brief overview of these related IoT communication technologies.

#### 1.1.1. IEEE802.15.4

IEEE 802.15.4 [4] is a standard specifying the physical layer and data link layer for Low-Rate Wireless Personal Area Networks (LR-WPANs). Supporting three un-licensed frequency bands (868 MHz, Europe; 928 MHz, North America; 2.4 GHz, worldwide), IEEE 802.15.4 can offer data rates up to 250 kbit/s at a transmission range largely dependent on the environment; while for a clear line-of-sight, up to 1000 m is possible; alas in most cases, the transmission range is measured in tenths of meters. Built on top of the IEEE 802.15.4 physical and data link layers, ZigBee [5] offers application-facing communications profiles and a network layer.

#### 1.1.2. Bluetooth/LE

Released in 1999 by a consortium led by Ericsson, Nokia and Intel, Bluetooth v1.0 was initially designed to, wirelessly, replace cables to connect devices typically used together, such as cell phones, laptops, headsets, keyboards, etc., offering a lower data rate (1-Mbps raw data rate, max) and a relatively short range (in theory, officially up to 100 m, at maximum transmission power, realistically, 5–10 m) while also a low power consumption.

Several revisions of Bluetooth later, Bluetooth 4.0 was completed in 2010. Fully compatible with Bluetooth 1.0, this revision supports a higher data rate (24-Mbps raw data rate, based on WiFi) and includes a “low energy” extension (called Bluetooth/LE or “Smart”). As compared with the “non-LE version”, Bluetooth/LE provides rapid link establishment functions (simpler pairing) and further trades off the data rate (approximately 200 kbps) for lower energy consumption, with the target to run a wireless sensor for at least one year on a single coin cell (approximately 200 *mAHr*) [6].

#### 1.1.3. IEEE 802.11 ah

IEEE [7,8] provides a wireless LAN standard that operates at sub-1-GHz license-exempt bands. The work is conducted by the IEEE 802.11 ah Task Group (TGah). Compared to IEEE 802.11 (operating at 2.4 GHz and 5 GHz), 802.11 ah supports a longer transmission range up to 1 km at the default transmission power of 200 mW. Depending on the bandwidth assigned, 802.11 ah can operate at 4 Mbps or 7.8 Mbps. If the channel condition is good enough, 802.11 ah can provide a hundreds of Mpbs data rate, thanks to the novel modulation and coding schemes brought from 802.11 ac.

#### 1.1.4. Sigfox

Sigfox (http://www.sigfox.com) is a variation of the cellular system that enables remote devices to connect to ab access point with Ultra Narrow Band (UNB). A proprietary technology, developed and delivered by the French company Sigfox, no detailed public specification is available. Sigfox operates on the 868-MHz frequency band, with the spectrum divided into 400 channels of 100 Hz [9]. Each end-device can send up to 140 messages per day, with a payload size of 12 octets, at a data rate up to 100 bps. Sigfox claims that each access point can handle up to a million end-devices, with a coverage area of 30–50 km in rural areas and 3–10 km in urban areas. Sigfox’s claim to being a low power technology stems, in no small part, from end-devices being heavily duty-cycled due to an assumption of the nature of the data traffic patterns in the IoT: when an end-device has a message to send, the Sigfox interface circuitry wakes up, and the message is transmitted “uplink”, from the end-device; then, the end-device listens for a short duration in case there are data being sent “downlink”, to the end-device. In other words, downlink traffic is supported by the end-device actively polling, which makes Sigfox an interesting choice for data acquisition, but perhaps less so for command-and-control scenarios.

#### 1.1.5. DASH7

DASH7 [10] is a wireless sensor and actuator full Open Systems Interconnection (OSI) stack protocol that operates in the 433-MHz, 868-MHz and 915-MHz unlicensed ISM band/SRD band. It originates from the ISO 18000-7 standard [11] for active RFID, intended by the U.S. Department of Defense for container inventory. DASH7 inherits from ISO/IEC 18000-7 the default parameters of the active air interface communication at 433 MHz, an asynchronous MAC and a presentation layer using highly structured data elements. Furthermore, DASH7 extends and defines the protocol stack from the physical layer up to the application layer.

DASH7 aims at providing communication in the range of up to 2 km, low latency, mobility support, multi-layer battery life, AES 128-bit shared key encryption support and a data rate up to 167 kbit/s.

In [12], a more detailed survey of different technologies, including 3GPP LTE Rel-13, Nokia’s narrow-band LTE-M, Neul/Huawei’s narrow-band proposal, Sigfox, etc., is provided.

### 1.2. Statement of Purpose

There have been a few articles related to LoRa in the literature. In [13,14], different long-range technologies, including LoRa, are compared. Petajajarvi et al. [15] studied the coverage of LoRa and proposed a channel attenuation model. In [16], the authors analyzed the LoRa capacity and proposed LoRaBlink to support multi-hop communications.

In complementing the work of these articles, the goal of this paper is three-fold: (i) given the semi-proprietary nature of LoRA (parts of the protocol are well documented; other parts are not), to provide an overview and functional description of LoRa and to present as much information as could be (experimentally and otherwise) gathered; (ii) to independently provide a quantification and evaluation of the performance of LoRA and of LoRaWAN, especially the spreading factor; and (iii) based on the analysis and performance evaluation, to propose possible solutions for performance enhancement.

The remainder of this paper is organized as follows: Section 2 provides a functional overview of LoRa, followed by Section 3, which describes and analyzes the LoRa physical layer in detail and provides experimental performance studies hereof. Following, the LoRaWAN MAC protocol is described in Section 4, with Section 5 presenting the evaluation hereof for LoRaWAN. Section 6 concludes this paper.

## 2. LoRa Overview

This section gives an overview of the LoRa protocol stack and basic network architecture.

### 2.1. LoRa Protocol Stack

LoRa, which stands for “Long Range”, is a long-range wireless communications system, promoted by the LoRa Alliance. This system aims at being usable in long-lived battery-powered devices, where the energy consumption is of paramount importance. LoRa can commonly refer to two distinct layers: (i) a physical layer using the Chirp Spread Spectrum (CSS) [17] radio modulation technique; and (ii) a MAC layer protocol (LoRaWAN), although the LoRa communications system also implies a specific access network architecture.

The LoRa physical layer, developed by Semtech, allows for long-range, low-power and low-throughput communications. It operates on the 433-, 868- or 915-MHz ISM bands, depending on the region in which it is deployed. The payload of each transmission can range from 2–255 octets, and the data rate can reach up to 50 Kbps when channel aggregation is employed. The modulation technique is a proprietary technology from Semtech.

LoRaWAN provides a medium access control mechanism, enabling many end-devices to communicate with a gateway using the LoRa modulation. While the LoRa modulation is proprietary, the LoRaWAN is an open standard being developed by the LoRa Alliance.

### 2.2. LoRa Network Architecture

**Figure 1:**
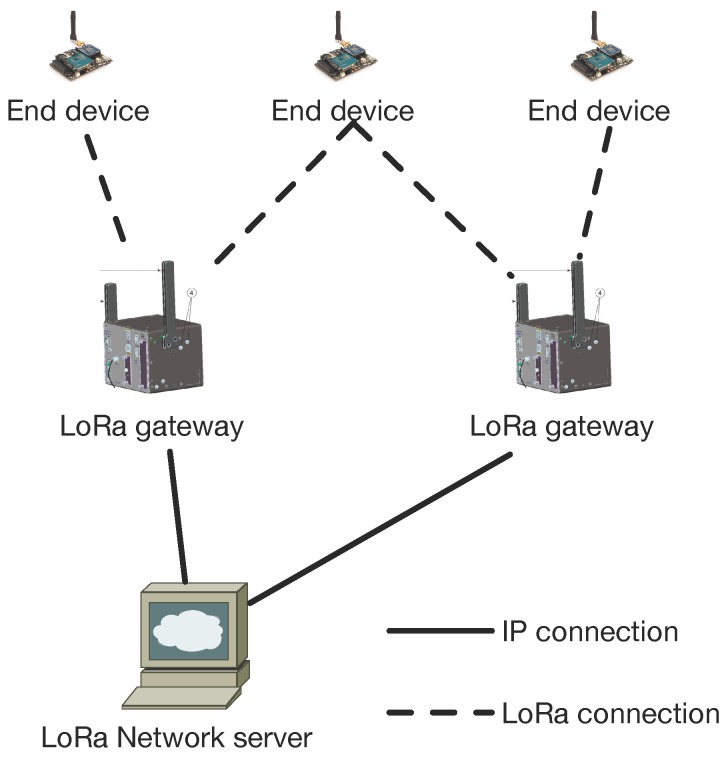
LoRa network architecture.

A typical LoRa network is “a star-of-stars topology”, which includes three different types of devices, as shown in Figure 1.

The basic architecture of a LoRaWAN network is as follows: end-devices communicate with gateways using LoRa with LoRaWAN. Gateways forward raw LoRaWAN frames from devices to a network server over a backhaul interface with a higher throughput, typically Ethernet or 3G. Consequently, gateways are only bidirectional relays, or protocol converters, with the network server being responsible for decoding the packets sent by the devices and generating the packets that should be sent back to the devices. There are three classes of LoRa end-devices, which differ only with regards to the downlink scheduling.

## 3. The LoRa Physical Layer

The LoRa modulation is a Semtech proprietary technology and is as such not fully open. This section presents an analysis (of the parts of LoRa that are open) and an experimental evaluation (of the proprietary parts of LoRa) with the purpose of understanding if the advertised performance of LoRa is observed in practice.

### 3.1. Overview of the Physical Layer

LoRa is a chirp spread spectrum modulation [18], which uses frequency chirps with a linear variation of frequency over time in order to encode information. Because of the linearity of the chirp pulses, frequency offsets between the receiver and the transmitter are equivalent to timing offsets, easily eliminated in the decoder. This also makes this modulation immune to the Doppler effect, equivalent to a frequency offset. The frequency offset between the transmitter and the receiver can reach 20% of the bandwidth without impacting decoding performance [19]. This helps with reducing the price of LoRa transmitters, as the crystals embedded in the transmitters do not need to be manufactured to extreme accuracy. LoRa receivers are able to lock on to the frequency chirps received, offering a sensitivity of the order of −130 dBm [19,20].

As the LoRa symbol duration is longer than the typical bursts of AMinterference generated by Frequency Hopping Spread Spectrum (FHSS) systems, errors generated by such interference are easily corrected through Forward Error-correction Codes (FECs). The typical out-of-channel selectivity (the maximum ratio of power between an interferer in a neighboring band and the LoRa signal) and co-channel rejection (the maximal ratio of power between an interferer in the same channel and the LoRa signal) of LoRa receivers is respectively 90 dB and 20 dB [19,20]. This outperforms traditional modulation schemes, such as Frequency-Shift Keying (FSK), and makes LoRa well suited to low-power and long-range transmissions.

### 3.2. Parameters of the Physical Layer

Several parameters are available for the customization of the LoRa modulation: Bandwidth (BW), Spreading Factor (SF) and Code Rate (CR). LoRa uses an unconventional definition of the spreading factor as the logarithm, in base 2, of the number of chirps per symbol. For the sake of simplicity, this article will stick to this definition. Theses parameters influence the effective bitrate of the modulation, its resistance to interference noise and its ease of decoding.

**Figure 2:**
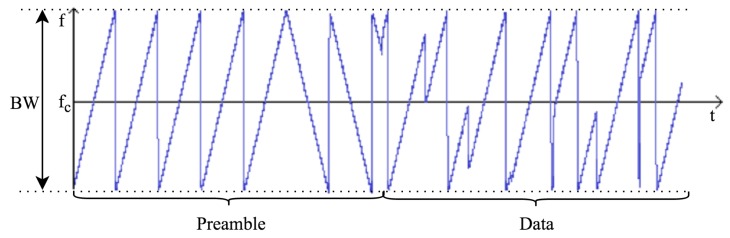
Frequency variation over time of a sample signal emitted by a LoRa transmitter. Data taken from [21]. $f_{c}$ is the central frequency of the channel, and BW is the bandwidth.

The bandwidth is the most important parameter of the LoRa modulation. A LoRa symbol is composed of $2^{SF}$ chirps, which cover the entire frequency band. It starts with a series of upward chirps. When the maximum frequency of the band is reached, the frequency wraps around, and the increase in frequency starts again from the minimum frequency. Figure 2 gives an example of a LoRa transmission in the frequency variation over time. The position of this discontinuity in frequency is what encodes the information transmitted. As there are $2^{SF}$ chirps in a symbol, a symbol can effectively encode SF bits of information.

In LoRa, the chirp rate depends only on the bandwidth: the chirp rate is equal to the bandwidth (one chirp per second per Hertz of bandwidth). This has several consequences on the modulation: an increase of one of the spreading factor will divide the frequency span of a chirp by two (as $2^{SF}$ chirps cover the whole bandwidth) and multiply the duration of a symbol by two, also. It will not, however, divide the bit rate by two, as one more bit will be transmitted in each symbol. Moreover, the symbol rate and the bit rate at a given spreading factor are proportional to the frequency bandwidth, so a doubling of the bandwidth will effectively double the transmission rate. This is translated in Equation (1), which links the duration of a symbol ($T_{S}$) to the bandwidth and the spreading factor.

(1) $$T_{S}=\frac{2^{SF}}{BW}$$

Moreover, LoRa includes a forward error correction code. The code rate (CR) equals 4/(4+n), with n∈{1,2,3,4}. Taking this into account, as well as the fact that SF bits of information are transmitted per symbol, the Equation (2) allows one to compute the useful bit rate ($R_{b}$).

(2) $$R_{b}=SF\times \frac{BW}{2^{SF}}\times CR$$

For example, a setting with BW=125 kHz, SF=7, CR=4/5 gives a bit rate of $R_{b}$ = 5.5 kbps.

These parameters also influence decoder sensitivity. Generally speaking, an increase of bandwidth lowers the receiver sensitivity, whereas an increase of the spreading factor increases the receiver sensitivity. Decreasing the code rate helps reduce the Packet Error Rate (PER) in the presence of short bursts of interference, i.e., a packet transmitted with a code rate of 4/8 will be more tolerant to interference than a signal transmitted with a code rate of 4/5. The figures in Table 1, taken from the SX1276 datasheet, are given as an indication.

Another parameter of the LoRa modulation, which is implemented in Semtech’s transceivers, is the low data rate optimization. This parameter is mandatory in LoRa when using spreading factors of 11 and 12 with a bandwidth of 125 kHz or lower. The effect of this parameter is not documented; however, Equation (3) shows that it reduces the number of bits transmitted per symbol by two.

### 3.3. Physical Frame Format

Although the LoRa modulation can be used to transmit arbitrary frames, a physical frame format is specified and implemented in Semtech’s transmitters and receivers. The bandwidth and spreading factor are constant for a frame.

A LoRa frame begins with a preamble. The preamble starts with a sequence of constant upchirps that cover the whole frequency band. The last two upchirps encode the sync word. The sync word is a one-byte value that is used to differentiate LoRa networks that use the same frequency bands. A device configured with a given sync word will stop listening to a transmission if the decoded sync word does not match its configuration. The sync word is followed by two and a quarter downchirps, for a duration of 2.25 symbols. The total duration of this preamble can be configured between 10.25 and 65,539.25 symbols. The structure of the preamble can be seen in Figure 2.

After the preamble, there is an optional header. When it is present, this header is transmitted with a code rate of 4/8. This indicates the size of the payload (in bytes), the code rate used for the end of the transmission and whether or not a 16-bit CRCfor the payload is present at the end of the frame. The header also includes a CRC to allow the receiver to discard packets with invalid headers. The payload size is stored using one byte, limiting the size of the payload to 255 bytes. The header is optional to allow disabling it in situations where it is not necessary, for instance when the payload length, coding rate and CRC presence are known in advance.

**Figure 3:**
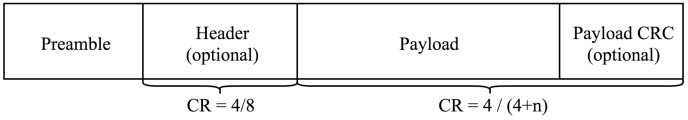
Structure of a LoRa frame. n∈{1..4}.

The payload is sent after the header, and at the end of the frame is the optional CRC. A schematic summarizing the frame format can be seen in Figure 3.

Equation (3), derived from Semtech’s datasheets [19,20], gives the number of symbols required to transmit a payload $n_{s}$, as a function of all of these parameters. This number should be added to the number of symbols of the preamble, in order to compute the total size of the packet in symbols. In this equation, PL is the payload size in bytes, CRC is 16 if the CRC is enabled and zero otherwise, *H* is 20 when the header is enabled and zero otherwise and DE is two when the low data rate optimization is enabled and zero otherwise. This equation also shows that the minimum size of a packet is eight symbols.

(3) $$n_{s}=8+max\left(\left\lceil \frac{8PL-4SF+8+CRC+H}{4\times (SF-DE)}\right\rceil \times \frac{4}{CR},0\right)$$

### 3.4. Performance Evaluation

**Figure 4:**
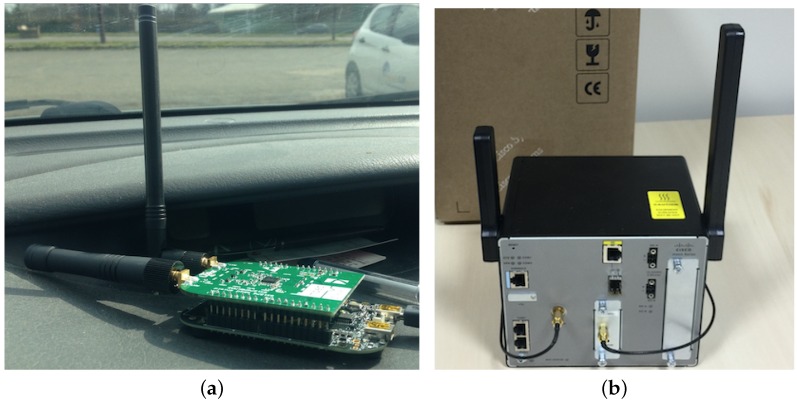
The LoRa testbed. (**a**) The LoRa end-device; (**b**) the LoRa gateway.

To verify whether the specified performance of LoRa receivers is reached in practice, a LoRa testbed is built. The Freescale KRDM-KL25Z development board with Semtech SX1276 MBED shield (Figure 4a) is used as the end-device, and a Cisco 910 industrial router is used as the gateway (Figure 4b). The gateway is connected to the network server provided by Thingpark (https://actility.thingpark.com) through Ethernet, so that the packet received can be monitored on the server side.

#### 3.4.1. Receiver Sensitivity

As there are many models and evaluations of the propagation of radio signals at the frequencies used by LoRa in various environments [22], this experiment is focused on checking the decoding performance of LoRa receivers.

**Figure 5:**
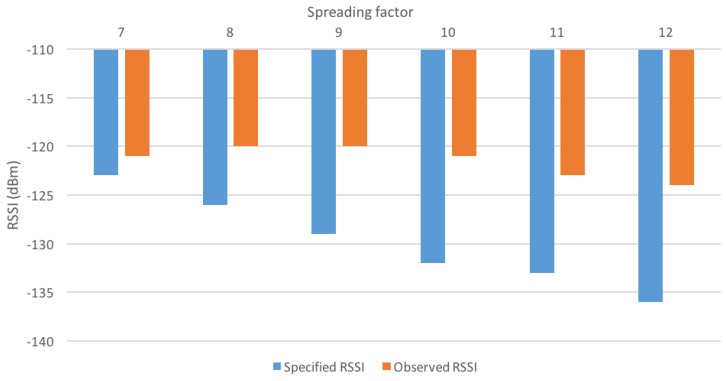
Minimal observed RSSIs with different spreading factors.

To this end, around 10,000 packets were sent from a LoRa device to the gateway, and the Received Signal Strength Indicators (RSSI) of received packets were recorded while moving the end-device. The gateway was placed indoors, and the device was outdoors, in an urban environment. All packets were sent with a bandwidth of 125 kHz and a code rate of 4/5. The transmit power of the device was set to the minimum (2 dBm, with a 3-dBi antenna) in order to limit the distance to cover before reaching low RSSIs. The order of magnitude of the distance between the end-device and the gateway at which packets started to get lost was 100 m. The minimal observed RSSIs are depicted in Figure 5.

These measured results are slightly above the specified values, and the expected decrease with the increase of the spreading factor is not observed. However, the packets achieving the lowest RSSIs were also received with a high SINR, close to 20 dB. This is likely due to the gateway being indoors, leading to additional shadowing.

It should be noted that the observed RSSIs are already 6 dB lower than the specified RSSIs when using FSK [19].

#### 3.4.2. Network Coverage

**Figure 6:**
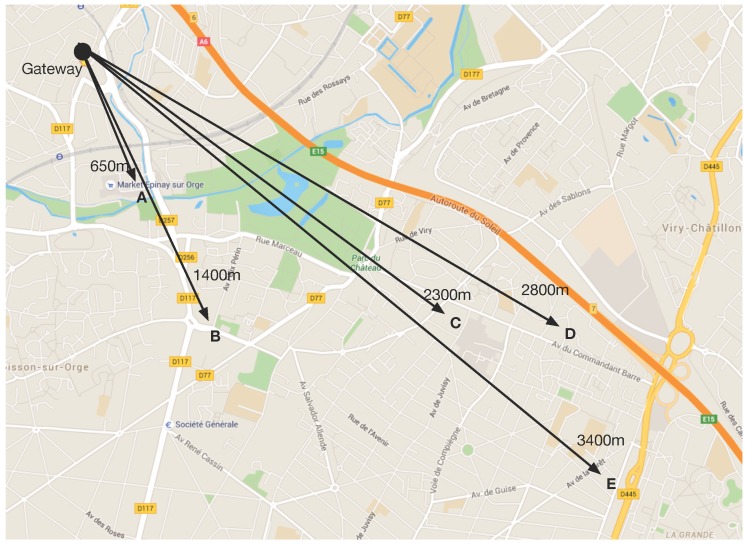
Map of LoRa field test.

This experiment aims at testing the network coverage of LoRa. Tests were conducted in a suburb of Paris, with mainly low-rise residential dwellings. The temperature was 15 °C, and the ambient humidity was 55%. The gateway was located on the second floor of a house, outside the window. Five different test points were chosen, with the distance to the gateway as shown in Figure 6. The end-device was in a car during the tests.

The transmission power of the end-device was set to 14 dBm, which is the default value as specified by [23]. To test the performance of different spreading factors, the packet acknowledgment and retransmission was turned off. The link check was also disabled so that the spreading factor will not change even if there is packet loss; by default, LoRa will adapt the spreading factor according to the link quality. Spreading factors of 7, 9 and 12 were chosen for the tests.

**Figure 7:**
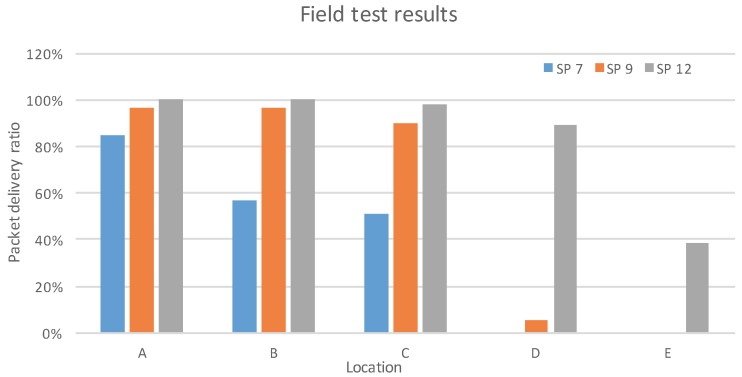
Packet delivery ratio of the LoRa field test.

Figure 7 shows the packet delivery ratio of different spreading factors with various distances. About 100 packets are transmitted to the network server in each test with a sequence number. The higher spreading factors have better coverage, as discussed in Section 3.2: for a spreading factor of 12, more than 80% of packets were received at Point D (2800 m), while no packet was received when using a spreading factor of seven. It is worth noting that the gateway was located in the second floor, which was about 5 m above the ground (normally, such a base station would be located at a higher altitude to achieve better coverage), and the test Point D was right behind a building of seven floors. The high delivery ratio using the high spreading factor has the cost of a much lower bit rate, as shown in Equation (2). On the other hand, the network coverage with low spreading factors is much lower.

It is important to note that the purpose of the tests above is to test the coverage of the LoRa physical layer using different spreading factors. In a real LoRa network with the LoRaWAN protocol, the end-devices are able to automatically increase the spreading factor if the transmission with the lower spreading factor fails. Furthermore, retransmission is also used if necessary. Therefore, in a network with LoRaWAN, a higher delivery ratio can be achieved.

## 4. The LoRaWAN Protocol

LoRaWAN is a MAC protocol, built to use the LoRa physical layer. It is designed mainly for sensor networks, wherein sensors exchange packets with the server with a low data rate and relatively long time intervals (one transmission per hour or even days). This section describes the LoRaWAN V1.0 specification [23], as released in January 2015.

### 4.1. Components of a LoRaWAN Network

Several components of the network are defined in the LoRaWAN specification and are required to form a LoRaWAN network: end-devices, gateways (i.e., base stations) and the network server.

- End-device: the low-power consumption sensors that communicate with gateways using LoRa.
- Gateway: the intermediate devices that forward packets coming from end-devices to a network server over an IP backhaul interface allowing a bigger throughput, such as Ethernet or 3G. There can be multiple gateways in a LoRa deployment, and the same data packet can be received (and forwarded) by more than one gateway.
- Network server: responsible for de-duplicating and decoding the packets sent by the devices and generating the packets that should be sent back to the devices.

Unlike traditional cellular networks, the end-devices are not associated with a particular gateway in order to have access to the network. The gateways serve simply as a link layer relay and forward the packet received from the end-devices to the network server after adding information regarding the reception quality. Thus, an end-device is associated with a network server, which is responsible for detecting duplicate packets, choosing the appropriate gateway for sending a reply (if any), consequently for sending back packets to the end-devices. Logically, gateways are transparent to the end-devices.

LoRaWAN has three different classes of end-devices to address the various needs of applications:

- Class A, bi-directional: Class A end-devices can schedule an uplink transmission based on their own needs, with a small jitter (random variation before transmission). This class of devices allows bi-directional communications, whereby each uplink transmission is followed by two short downlink receive windows. Downlink transmission from the server at any other time has to wait until the next uplink transmission occurs. Class A devices have the lowest power consumption, but also offer less flexibility on downlink transmissions.
- Class B, bi-directional with scheduled receive slots: Class B end-devices open extra receive windows at scheduled times. A synchronized beacon from the gateway is thus required, so that the network server is able to know when the end-device is listening.
- Class C, bi-directional with maximal receive slots: Class C end-devices have almost continuous receive windows. They thus have maximum power consumption.

It should be noted that LoRaWAN does not enable device-to-device communications: packets can only be transmitted from an end-device to the network server, or vice-versa. Device-to-device communication, if required, must thus be sling-shot through the network server (and consequently, by way of two gateway transmissions).

The LoRaWAN specification states that LoRaWAN networks should use ISM frequency bands. These bands are subject to regulations regarding the maximum transmission power and the duty cycle. These duty cycle limitations translate into delays between the successive frames sent by a device. If the limitation is at 1%, the device will have to wait 100-times the duration of the last frame before sending again in the same channel.

### 4.2. LoRaWAN Message Format

LoRaWAN uses the physical frame format described in Section 3.3. The header and CRC are mandatory for uplink messages, which makes it impossible to use a spreading factor of six in LoRaWAN. Downlink messages have the header, but not the CRC. The code rate that should be used is not specified and neither is when the end-devices should use the low data rate optimization.

**Figure 8:**
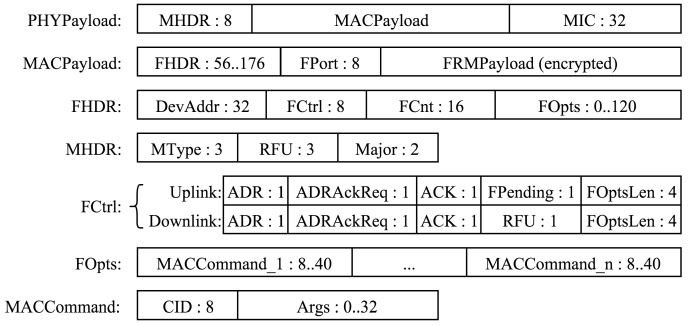
LoRaWAN frame format. The sizes of the fields are in bits.

The message format is detailed in Figure 8. *DevAddr* is the short address of the device. *FPort* is a multiplexing port field. The value zero means that the payload contains only MAC commands. When this is the case, the *FOptsLen* field must be zero. *FCnt* is a frame counter. *MIC* is a cryptographic message integrity code, computed over the fields *MHDR*, *FHDR*, *FPort* and the encrypted *FRMPayload*. *MType* is the message type, indicating among other things whether it is an uplink or a downlink message and whether or not it is a confirmed message. Acknowledgments are requested for confirmed messages. *Major* is the LoRaWAN version; currently, only a value of zero is valid. *ADR* and *ADRAckReq* control the data rate adaptation mechanism by the network server. *ACK* acknowledges the last received frame. *FPending* indicates that the network server has additional data to send and that the end-device should send another frame as soon as possible so that it opens receive windows. *FOptsLen* is the length of the *FOpts* field in bytes. *FOpts* is used to piggyback MAC commands on a data message. *CID* is the MAC command identifier, and *Args* are the optional arguments of the command. *FRMPayload* is the payload, which is encrypted using AES with a key length of 128 bits. The minimal size of the MAC header is 13 bytes; its maximal size is 28 bytes. Knowing this, it is possible to compute the maximum channel capacity available for application data payloads with given modulation parameters thanks to Equations (1) and (3). As packets are sent from a device to the network server and vice versa, there is no destination address on uplink packets, and there is no source address on downlink packets.

### 4.3. End-Device Setup

In order to participate in a LoRaWAN network, an end-device must be activated. LoRaWAN provided two ways to activate an end-device: Over-The-Air Activation (OTAA) and Activation By Personalization (ABP).

The activation process should give the following information to an end-device:

- End-device address (*DevAddr*): A 32-bit identifier of the end-device. Seven bits are used as the network identifier, and 25 bits are used as the network address of the end-device.
- Application identifier (*AppEUI*): A global application ID in the IEEE EUI64 address space that uniquely identifies the owner of the end-device.
- Network session key (*NwkSKey*): A key used by the network server and the end-device to calculate and verify the message integrity code of all data messages to ensure data integrity.
- Application session key (*AppSKey*): A key used by the network server and end-device to encrypt and decrypt the payload field of data messages.

For OTAA, a join procedure with a join-request and a join-accept message exchange is used for each new session. Based on the join-accept message, the end-devices are able to obtain the new session keys (*NwkSkey* and *AppSKey*). For the ABP, the two session keys are directly stored into the end-devices.

### 4.4. LoRaWAN MAC Commands

LoRaWAN defines many MAC commands that allow customizing end-device parameters [23]. One of them, *LinkCheckReq*, can be sent by an end-device to test its connectivity. All of the others are sent by the network server. These commands can control the data rate and output power used by the device, as well as the number of times each unconfirmed packet should be sent (*LinkADRReq*), the global duty cycle of the device (*DutyCycleReq*), changing parameters of the receive windows (*RXTimingSetupReq*, *RXParamSetupReq*) and changing the channels used by the device (*NewChannelReq*). One command is used to query the battery level and reception quality of a device (*DevStatusReq*).

## 5. LoRaWAN Analysis

This section analyzes and discusses the performance of LoRaWAN by way of experiments and simulations. As in the previous section, all of this study is based on [23].

### 5.1. Single Device Maximal throughput and MTU

**Figure 9:**
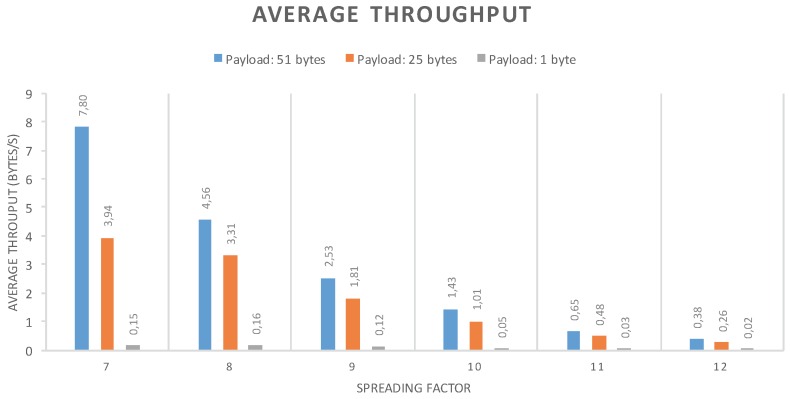
Maximum throughput attained by a single device using LoRaWAN.

The goal of this experiment is to evaluate the maximal throughput that a single device can obtain. This depends more on the physical layer than on the MAC protocol, but it gives an idea of what is possible when using LoRaWAN. The experiment was conducted by having a device send data as soon as the channel limitations and the protocol allow it. Tests were conducted with six channels of 125 kHz and using spreading factors from 7–12. No MAC commands were sent, so the size of the MAC header was always 13 bytes. The results, depending on the payload size, are visible in Figure 9, which are measured over about 100 packets transmitted in each test. Fifty-one bytes are the maximum payload size allowed by the implementation used for the tests.

This experiment revealed that at low packet sizes, the limiting factor was not the channel duty cycle limitations, as could have been expected, but the duration of the receive windows. Indeed, the device has to wait for the two downlink receive windows following the transmission to be over before sending another packet. However, this situation is not the use case LoRaWAN was designed for: the goal of LoRaWAN is rather to manage large quantities of devices that send a few bytes of data from time to time.

In the tests above, the MAC header is always 13 bytes. However, in practice, the LoRaWAN header can be a variable size between 13 and 28 bytes. Moreover, the maximum size of the frame depends on the data rate used [23], and LoRaWAN does not have a mechanism to split large payloads over multiple frames. As of the current specification, the application above LoRaWAN has no way of knowing what the maximal size of the packet that it will be able to send in the next transmission is, which might be problematic. A conservative approach is to never try to send more than the smallest maximum payload size, which is 36 bytes, but this results in a loss of capacity if a large amount of data has to be sent, as well as lower throughput, as shown in the results in Figure 9. This is relatively easy to address in a future LoRaWAN specification revision, either by adding a fragmentation mechanism or by informing the upper layer of the MTU from MAC protocol.

### 5.2. Total Capacity and Channel Load

The total capacity of the network is not only related to payload size. As two transmissions on the same frequency, but at different spreading factors, can be decoded simultaneously, in what follows, a logical channel is defined by a pair (frequency band, spreading factor).

The total transmission capacity of a LoRaWAN network is the sum of the capacities of all of the logical channels. In a 125-kHz frequency band, there are six possible spreading factors (from 7 to 12), which brings the total capacity of a 125-kHz channel to 12,025 bps.

In the EU frequency band, the set of mandatory channels contains three 125-kHz channels [23], which make the minimum total capacity of the network 36 kbps. Networks operators are free to add more channels (sent to the devices using *NewChannelReq* commands), thus increasing the capacity of the network.

As the transmission bit rate is dependent on the spreading factor, not all logical channels have the same capacity. In what follows, the load for a logical channel is defined by the time average of the numbers of LoRa devices trying to send data. This coincides with the natural definition of the load: in optimal conditions, i.e., with a perfect synchronization of the devices, a load of one can be reached, saturating the channel.

### 5.3. Estimation of the Collision Rate

As of the current specification, the devices and the gateways can transmit at any time. There is no listen-before-talk or CSMAmechanism. This makes LoRaWAN very similar to ALOHA [24], but contrary to ALOHA with a variable packet length.

Because of the legal duty-cycle limitations of 1% in the EU region where this analysis took place, 100 devices would have been needed to emulate a load of one, and this number would have grown proportionally to the maximum link load we wanted to test. As there were not this many devices on hand, simulations are used to evaluate LoRaWAN’s behavior under load.

A simulator was built to simulate the random process of packet emissions. Five-hundred-thousand packets were simulated for each data point. If the transmission time of two packets overlaps, we consider that a collision happens and that none of the two packets reaches the gateway. The collision rate is the number of packets that collided, divided by the total number of packets sent during the simulation. The channel capacity usage is computed as the amount of data that is successfully transferred during the simulation, divided by the theoretical maximum amount of data that could have been sent in the channel, which is the channel capacity multiplied by the simulation duration. The channel load is as defined in the previous Section 5.2, or equivalently, the sum of the duration of all of the packets sent during the simulation, divided by the duration of the simulation.

The duration of the packets for the different payload sizes was computed using Semtech’s LoRa Calculator, for a spreading factor of seven, a bandwidth of 125 kHz, a code rate of 5/4 and six symbols in the preamble.

**Figure 10:**
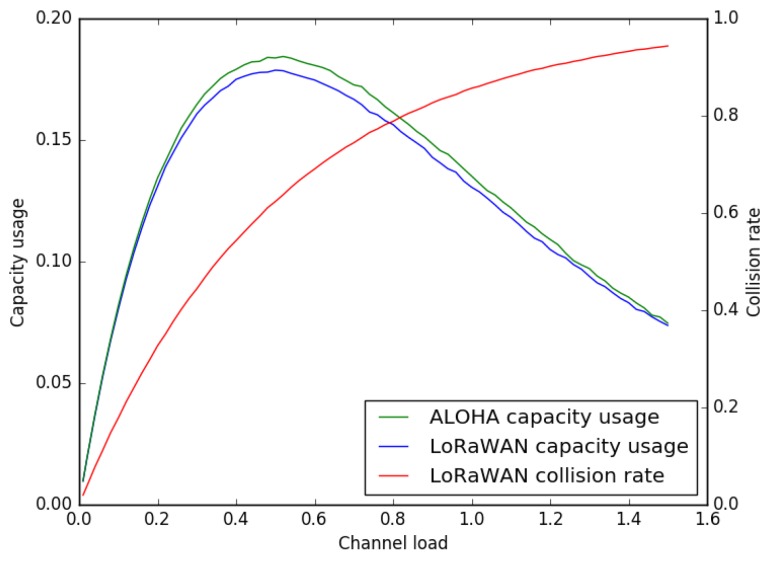
Link capacity usage and packet collision rate for a LoRaWAN network and compared to an ALOHA network. The load is as defined in Section 5.2.

Assuming the packet arrivals are following a Poisson law and a uniform distribution of the payloads lengths between one and 51 bytes, the expected capacity usage and collision rate depending on the load for one logical channel can be plotted. The result is shown in Figure 10.

The variable packet length does not greatly impact the performance of LoRaWAN, and all said and done, the observed behavior is very close to that of pure ALOHA. The maximum capacity usage is 18% of the channel capacity and is reached for a link load of 0.48. However, at this load, around 60% of the packets transmitted are dropped because of collisions.

This may be an issue, because if the devices are not using confirmed messages, some messages will be lost (and increasing the number of times each message is sent by the devices is a bad solution, as it will increase the load on the link), and if the devices are using confirmed messages, they will have to retransmit most packets several times, which will in addition impact the battery life of the devices.

LoRaWAN confirmed messages sent by the devices must be acknowledged by a packet sent during one of the two receive windows following the transmission, while confirmed messages sent by the gateway will be acknowledged during the next uplink transmission. The acknowledgment is only a flag in the packet header, and the setting of this flag acknowledges the last message received. As such, when using confirmed messages, a new packet should not be sent before the acknowledgment of the previous packet was received; otherwise, it will be impossible to know to which packet the next acknowledgment will be referring.

**Figure 11:**
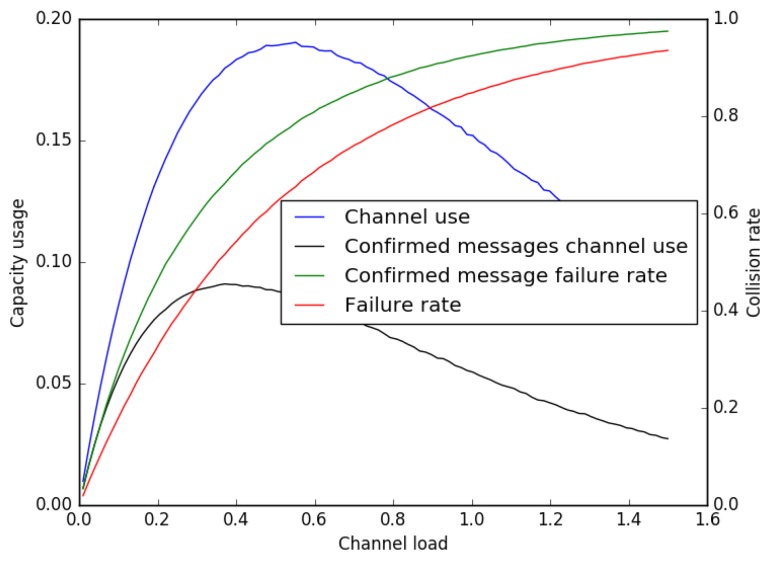
Link capacity usage and packet collision rate for a LoRaWAN network when using confirmed messages. The load is as defined in Section 5.2.

The drawback of this mechanism is that a confirmed message requires two successive transmissions in order to be successful, thus increasing the collision probability with other messages and the number of retransmissions needed. As above, the probability of success and the link capacity usage when end-devices are sending confirmed messages can be plotted. For this simulation, we consider that the gateway does not send MAC commands to the device, so the acknowledgment message is always using a 13-byte MAC header and no payload. We also take these messages into account in the computation of the load, i.e., when the sum of the durations of all of the messages and their acknowledgments is equal to the duration of the simulation, the value of the load is one. The result is shown in Figure 11.

As expected, the success rate is significantly lower than without confirmed messages. However, this is a relatively efficient way of implementing this functionality, because two successful transmissions are necessary anyway.

The results show that LoRaWAN is extremely sensitive to the channel load, similar to ALOHA. The solution implemented by usual network protocols, such as 802.11 or cellular networks, to help mitigate this problem is CSMA [25]. In order to ensure the scalability of LoRaWAN, it could be interesting to study the feasibility of the implementation of a CSMA mechanism into LoRaWAN. A possible issue is the duty cycle limitation that applies to the gateway, and that would prevent it from sending messages too often; another is the potential non-transitivity of the channel (i.e., an end-device may or may not be able to “carrier sense” if another end-device is transmitting to the same gateway). If the current architecture is kept, the CSMA mechanism would have to be controlled by the network server, which would put even more load on it. Alas, a CSMA mechanism could also remove the risk of collision of the acknowledgment for confirmed messages, by making it happen during a contention-free period.

The current LoRaWAN specification does not have any means to enforce quality of service, and thus, it should not be used for critical applications or applications where the delay between the first time at which the device tries to send a message and the time at which it is received is important. Adjusting the number of times a device sends its packets may increase the chance of these packets going through, but it does so at the expense of more collisions with transmissions from other nodes and does not provide any hard guarantee.

LoRaWAN currently uses ISM bands, which have the advantage of being free and not requiring a license. However, these bands are more and more used by LoRaWAN’s competitors. Even if LoRa is very resistant to interferences, these bands have a finite capacity, and it is not guaranteed that the capacity of this band is sufficient. Moreover, it is perfectly legal for a malicious individual to emit random LoRa symbols, which will jam LoRa transmissions. Using a proprietary frequency band would have the advantage to remove most interferences, as well as remove the duty cycle cap, possibly making the implementation of a CSMA mechanism easier.

### 5.4. The Network Server Role

LoRaWAN specifies the behavior of the devices, but not the behavior of the network server. As shown in Section 5.3, it is important to keep the load on the network low, and the network server has to enforce this by sending MAC commands to the devices. However, as this is not part of the specification and as there is no open source reference implementation (as of the writing of this article), a correct behavior of the network server is hard to be evaluated.

The network server can easily degrade the performance of the network. For instance, it can use the *LinkADRReq* command to configure the number of times a device will send each data frame. This parameter is advertised as a way to control the quality of service for a device. Setting this parameter to more than one will increase the load on the network, increasing the amount of collisions, and thus, should be done very cautiously.

Moreover, LoRaWAN networks are advertised to be able to handle millions of devices. The network server will be responsible for the optimization of all of these nodes. Even if the event rate in sensor networks is significantly lower than in traditional networks, the performance of the network server should be carefully evaluated by the network operators, to ensure the scaling of the network.

### 5.5. The Gateway Role

The current specification states that the gateway is only a relay. This is linked to the fact that the packets sent by the devices have no destination address (which saves a few bytes) and that there is no association between a device and a gateway. Indeed, as several gateways can receive the same message from a device, only one of them should reply to it. It falls back to the network server to choose the best gateway.

The only task that should be handled by the gateways is the timing of the downlink messages. This timing should be accurate so that the device receives the message in its receive window. It is not specified whether the gateways receive a message to send from the server along with the time at which it should be sent or if the gateway sends the message received from the server as soon as it receives it, and it is unclear which solution is implemented in existing gateways. As the round trip time of the backhaul interface of the gateways cannot be controlled, the first solution should be implemented. It would also allow one to synchronize the transmissions of the different gateways, avoiding collisions between them.

**Figure 12:**
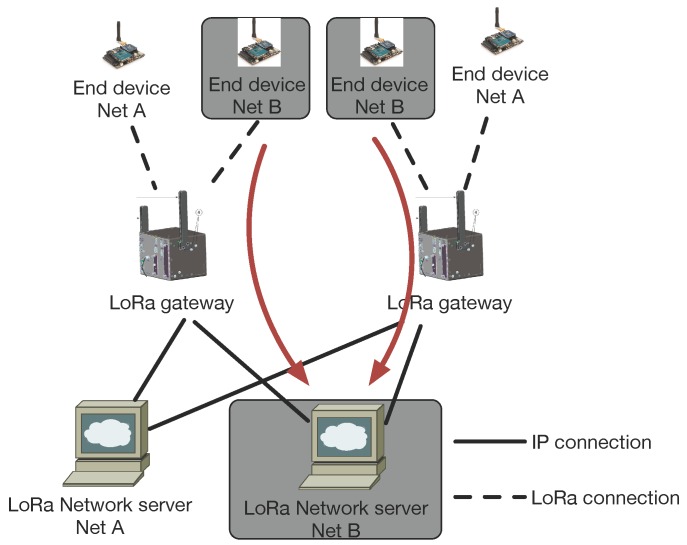
An example of shared gateways in LoRa. The gateways can forward the packet to different network servers.

In the current specification, each gateway is dedicated to a specific network server, as shown in Figure 1. This means both the gateways and the data collected are “owned” by the entity that runs the only network server. In the future, it would be interesting to extend the function of the gateways so that they can forward the packet to specific network servers, as shown in Figure 12. This may effectively reduce the expense of devices and network deployment.

## 6. Conclusions

LoRa is a long-range and low-power telecommunication systems for the “Internet of Things”. The physical layer uses the LoRa modulation, a proprietary technology with a MAC protocol. LoRaWAN is an open standard with the specification available free of charge [23].

This paper gives a comprehensive analysis of the LoRa modulation, including the data rate, frame format, spreading factor, receiver sensitivity, etc. A testbed has been built, to experimentally study the network performance, documented in this paper . The results show that LoRa modulation, thanks to the chirp spread spectrum modulation and high receiver sensitivity, offers good resistance to interference. Field tests show that LoRa can offer satisfactory network coverage up to 3 km in a suburban area with dense residential dwellings. The spreading factor has significant impact on the network coverage, as does the data rate. LoRa is thus well suited to low-power, low-throughput and long-range networks.

This paper has also shown that LoRaWAN is an LPWAN protocol very similar to ALOHA. Its performance thus degrades quickly when the load on the link increases.

## Figures and Tables

**Table 1:** Semtech SX1276 LoRa receiver sensitivity in dBm at different bandwidths and spreading factors, taken from [19].

	SF	7	8	9	10	11	12
BW							
125 kHz		−123	−126	−129	−132	−133	−136
250 kHz		−120	−123	−125	−128	−130	−133
500 kHz		−116	−119	−122	−125	−128	−130
